# 

**Published:** 1914-06-20

**Authors:** 


### Gifts and Bequests.

MR. ROBERT HENRY OTTER, M.A., of Chertsey, Surrey, and of Sugworth, Yorks, has left £200 each to the Bristol Royal Infirmary, the Bristol General Hospital, the Lincoln County Hospital, the Brompton Hospital for Consumption, the Ventnor Hospital for Consumption, and the Surrey County Hospital, Guildford; and £100 each to the Surrey Convalescent Home for Men, Seaford; the Victoria Convalescent Home for Surrey Women, Bognor; the Convalescent Home for Surrey Children, Bognor; the Alexandra Sanatorium for Consumption, Davos Platz, Switzerland; and the Bristol Hospital for Women and Children.

### The Book Market.

LIST OF NEW BOOKS RECEIVED FROM THE FOLLOWING PUBLISHERS:—

Geo. Allen: "One Man's Way." By Evelyn Dickinson. 6s. net.

A. and C. Black, Ltd.: "The Social Guide, 1914." 2s. 6d. net.

Cassell and Co.: "Tropical Diseases." By Sir Patrick Manson. Fifth Edition. 12s. 6d. net.

J. and A. Churchill: "Apsley Cookery Book." By Mrs. John J. Webster and Mrs. H. Llewelyn. 3s. 6d. net.

F. Gittler (Paris): "Physiologie Normale et Pathologique des Reins," by L. Ambard; "Notions Pratiques d'Electrothérapie Appliquée à l'Urologie," by Dr. Denis Courtade; "Manuel de Cystoscopie," by E. Papin.

Wm. Heinemann: "The Pocket Formulary for the Treatment of Disease in Children." By J. P. Ludwig, M.D., Vienna. Fourth Edition. 7s. 6d. net.

### Institution and Public Reports.

Annual Report (for 1913) of the Brighton, Hove, and Preston Dispensary.

One Hundred and Sixty-ninth Annual Report (for 1913) of the Worcester General Infirmary.

### Pamphlets and Papers.

A Short History of Worcester General Infirmary (October 13), by Alderman H. Leicester.

Historical Sketch of the London Homœopathic Hospital. Published for distribution at the Hospital, Great Ormond Street and Queen Square.

RATES OF SUBSCRIPTION: Twelve months, 6/6; Six months, 4/-; Three months, 2/-, post free to any address in the United Kingdom. Abroad, Twelve months, 8/8; Six months, 5/-; Three months, 2/6, post free.—Address The Subscription Dept., "THE HOSPITAL," The Hospital Building, 28/29 Southampton Street Strand, London, W.C.

**28/29 SOUTHAMPTON STREET, STRAND, LONDON, W.C.**

